# A Clinical Study Provides the First Direct Evidence That Interindividual Variations in Fecal β-Lactamase Activity Affect the Gut Mycobiota Dynamics in Response to β-Lactam Antibiotics

**DOI:** 10.1128/mbio.02880-22

**Published:** 2022-11-30

**Authors:** Margot Delavy, Charles Burdet, Natacha Sertour, Savannah Devente, Jean-Denis Docquier, Nathalie Grall, Stevenn Volant, Amine Ghozlane, Xavier Duval, France Mentré, Christophe d’Enfert, Marie-Elisabeth Bougnoux

**Affiliations:** a Institut Pasteur, Université Paris Cité, INRAE USC2019, Unité Biologie et Pathogénicité Fongiques, Paris, France; b Université Paris Cité, IAME, INSERM, Paris, France; c AP-HP, Département d’Epidémiologie, Biostatistique et Recherche Clinique, Hôpital Bichat, Paris, France; d Università di Siena, Dipartimento di Biotecnologie Mediche, Siena, Italy; e Institut Pasteur, Université Paris Cité, Bioinformatics and Biostatistics Hub, Paris, France; f Clinical investigation center, INSERM 1425, IAME, Hôpital Bichat, Assistance Publique des Hôpitaux de Paris (APHP), Université Paris Cité, Paris, France; g Unité de Parasitologie-Mycologie, Service de Microbiologie Clinique, Hôpital Necker-Enfants-Malades, Assistance Publique des Hôpitaux de Paris (APHP), Paris, France; Duke University

**Keywords:** antibiotics, *Candida albicans*, gut mycobiota, healthy individuals, beta-lactamases

## Abstract

Antibiotics disturb the intestinal bacterial microbiota, leading to gut dysbiosis and an increased risk for the overgrowth of opportunistic pathogens. It is not fully understood to what extent antibiotics affect the fungal fraction of the intestinal microbiota, the mycobiota. There is no report of the direct role of antibiotics in the overgrowth in healthy humans of the opportunistic pathogenic yeast Candida albicans. Here, we have explored the gut mycobiota of 22 healthy subjects before, during, and up to 6 months after a 3-day regimen of third-generation cephalosporins (3GCs). Using ITS1-targeted metagenomics, we highlighted the strong intra- and interindividual diversity of the healthy gut mycobiota. With a specific quantitative approach, we showed that C. albicans prevalence was much higher than previously reported, with all subjects but one being carriers of C. albicans, although with highly variable burdens. 3GCs significantly altered the mycobiota composition and the fungal load was increased both at short and long term. Both C. albicans relative and absolute abundances were increased but 3GCs did not reduce intersubject variability. Variations in C. albicans burden in response to 3GC treatment could be partly explained by changes in the levels of endogenous fecal β-lactamase activity, with subjects characterized by a high increase of β-lactamase activity displaying a lower increase of C. albicans levels. A same antibiotic treatment might thus affect differentially the gut mycobiota and C. albicans carriage, depending on the treated subject, suggesting a need to adjust the current risk factors for C. albicans overgrowth after a β-lactam treatment.

## INTRODUCTION

Interest in the role of the gut microbiota in health and disease is rising (1–4) and the role of antibiotics as major disturbers of the microbiota healthy state has been largely studied (5–8). By killing the resident bacteria of the gut, broad-spectrum antibiotics reduce bacterial diversity in the gastrointestinal (GI) tract and decrease the abundance of beneficial bacteria (5, 7). They also alter the gut microbiota interaction network, thus contributing to the overgrowth of opportunistic pathogens (6, 9). More alarmingly, the prolonged use of antibiotics may promote antibiotic resistance (10). For example, β-lactam exposure can lead to the selection of specific gut bacteria able to produce β-lactamases, enzymes that can hydrolyze β-lactam antibiotics, leading to an overall increase in antibiotic resistance (11, 12).

While the bacterial microbiota is extensively studied, less attention has been paid to the mycobiota—the fungal part of the microbiota—and to the consequences that antibiotic-induced dysbiosis may have on the fungal communities of the gut. It is now well established that fungi can rapidly proliferate in the GI tract of mice after removal of gut bacteria by antibiotics (13). The GI tract of mice is not naturally colonized by the opportunistic pathogen Candida albicans and antibiotics have been used to trigger such colonization (14), suggesting that they clear specific bacteria able to inhibit C. albicans growth in the mouse GI tract (15). Yet, we need more information about the impact of an antibiotic-induced dysbiosis on the healthy human gut mycobiota and specifically C. albicans. Because C. albicans systemic infections are responsible for thousands of deaths each year (16) and antibiotics are a well-known risk factor for these infections (17), we need to better understand the mechanisms of C. albicans overgrowth in the human gut, upon antibiotic treatment.

In this work, we prospectively followed two parallel groups of 11 healthy subjects each, before, during, and after they were treated intravenously with either cefotaxime or ceftriaxone, two third-generation cephalosporin (3GC) antibiotics that share a similar activity spectrum (8). We quantified the levels of C. albicans carriage in all subjects and characterized their healthy mycobiota and its variability during the 2-week period preceding antibiotic administration. Then, we analyzed the changes in terms of fungal diversity, fungal burden, community profile, and C. albicans levels, occurring in the mycobiota after antibiotics were administered, both at short and long term. Finally, we monitored the level of fecal β-lactamase activity, which is known to modulate the intensity of the post-3GC intestinal dysbiosis, and we correlated the changes in β-lactamase activity with the impact of 3GCs on C. albicans carriage.

## RESULTS

### The gut mycobiota of healthy subjects is highly dynamic and variable.

To study the healthy mycobiota, we used fecal samples collected from each of 22 healthy volunteers at day −15 (−D15), −D7, and −D1, before antibiotic administration (see Materials and Methods). In total, 54 fecal samples (one to three available per subject) were available for analyses.

First, we assessed the fungal load, i.e., the ratio between the fungal DNA concentration and the total fecal DNA (see Materials and Methods). The mycobiota represented a very small fraction of the total microbiota in healthy subjects (median fungal load: 7.9 × 10^−6^, min: 6.7 × 10^−10^, max: 1.5 × 10^−3^, Fig. S1A).

10.1128/mbio.02880-22.1 FIG S1 Phyla and genera composition of the healthy mycobiota in 22 healthy subjects during a 2-weeks period. (A) Violin plot of the fungal load (fungal 18S rRNA DNA relative to the total fecal DNA) at 1-week apart time points for 22 healthy subjects. (B) Barplots of the average relative abundance of the main fungal phyla at 1-week apart time points for 22 healthy subjects. (C) Barplots of the average relative abundance of the main fungal genera at 1-week apart time points for 22 healthy subjects. Download FIG S1, TIF file, 1.6 MB. Copyright © 2022 Delavy et al. 2022 Delavy et al. https://creativecommons.org/licenses/by/4.0/ This content is distributed under the terms of the Creative Commons Attribution 4.0 International license.

Using ITS1 sequencing, we further characterized the mycobiota composition of the 22 subjects during the 2 weeks preceding 3GC exposure. We identified 233 different OTUs, 182 OTUs (78.1%) being annotated at the phylum level, 167 (71.7%) at the genus level, and 123 (52.8%) at the species level. Overall, the 167 OTUs annotated at the genus level and the 123 OTUs annotated at the species level represented 99.7% and 91.2% of the total number of sequences, respectively.

**FIG 1:**
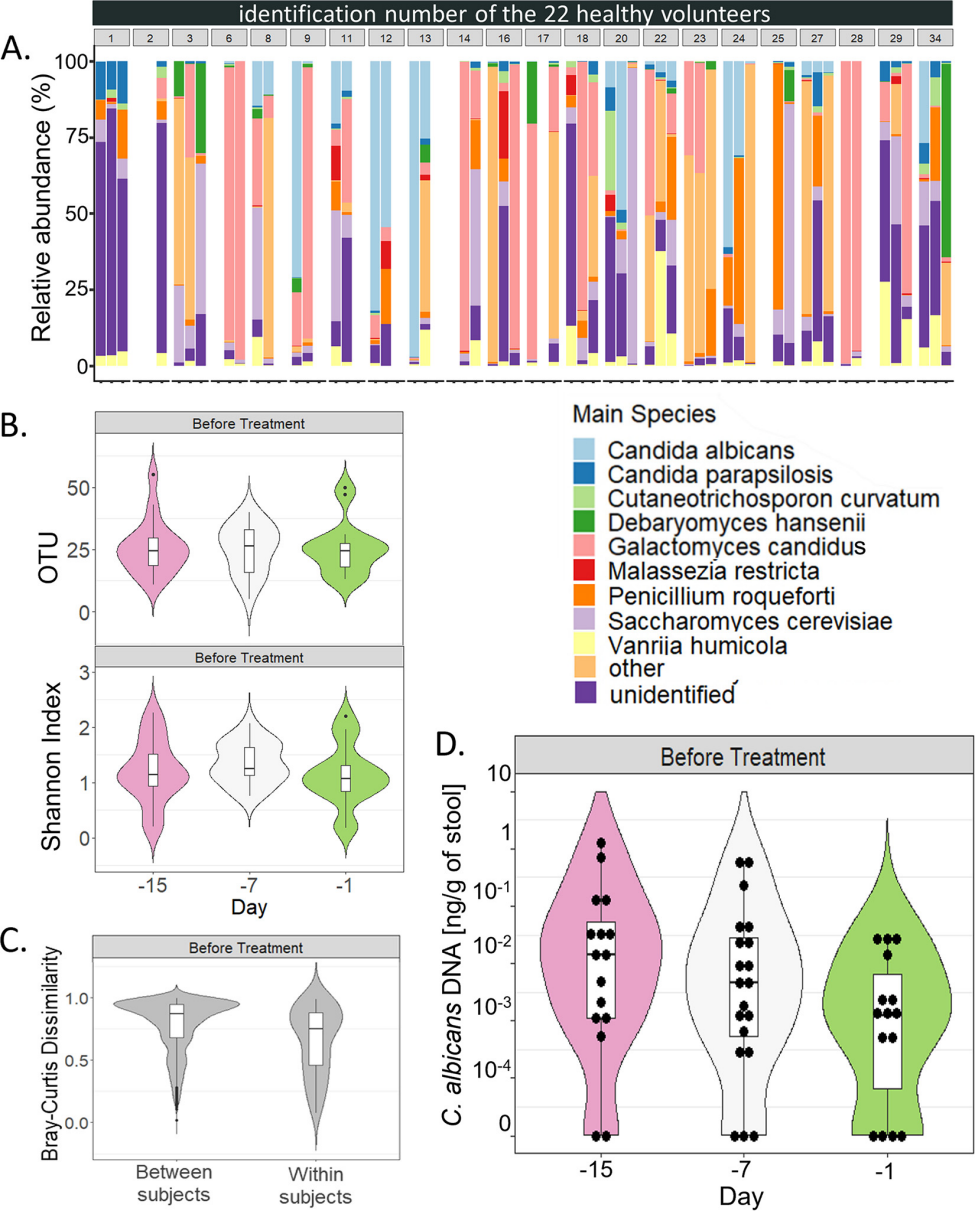
Dynamic of the mycobiota characteristics in 22 healthy individuals during a 2-week period. (A) Fungal species relative abundances at 1-week apart time points for 22 healthy subjects. For each subject, barplot are ordered by time (−D15, −D7, −D1 before antibiotics). Represented species reached a mean relative abundance across subjects above 1%. (B) Alpha diversity: violin plot of the number of OTUs and of the Shannon index values at 1-week apart time points for 22 healthy subjects. (C) Beta diversity: Bray-Curtis dissimilarity values between samples donated by different subjects (between subjects) and between samples donated by the same subjects (within subjects) for ITS1 sequencing data. Values range from 0 to 1, with 0 being the least dissimilar and 1 being the most dissimilar. (D) Violin and boxplots of the C. albicans DNA levels at 1-week apart time points for 22 healthy subjects. Each dot represents a sample. For all panels, the upper whiskers extend from the hinge to the largest value below 1.5× the interquartile range, and the lower whiskers extend from the hinge to the smallest value above 1.5× the interquartile range.

Ascomycota was the most abundant phylum (mean relative abundance of 77.9%), followed by Basidiomycota (21.9%; Fig. S1B). Sixty-two fungal genera were identified in at least two samples, with eight reaching a mean relative abundance across subjects above 1% (Fig. S1C). Ninety-five species were identified in at least two samples, nine reaching a mean relative abundance across subjects above 1% (Table 1). The taxa relative abundances were highly variable between individuals and across time (Fig. 1A), with *Galactomyces candidus* being the most disparately represented taxa, with a relative abundance varying from 0% to 99.2% depending on the sample.

**TABLE 1:** Prevalence of the main fungal species in healthy subjects and in their fecal samples, estimated by ITS1 sequencing

Main fungal species	Prevalence^*a*^	
	Fecal samples (%) (*N* = 54)	Healthy subjects (%) (*N* = 22)
*Vanrija humicola*	98.2	100.0
*Galactomyces candidus*	92.6	95.5
Saccharomyces cerevisiae	88.9	95.5
Candida parapsilosis	88.9	95.5
*Penicillium roqueforti*	72.2	90.9
*Cutaneotrichosporon curvatum*	87.0	86.4
*Malassezia restricta*	88.8	77.3
Candida albicans	75.9	72.7
Debaryomyces hansenii	68.5	59.1

a A species is considered present in a sample if its relative abundance is above 0.1%. A species is considered present in a subject if it is present in at least one sample between −D15 and −D1.

We identified a median of only 25 OTUs per sample (min: 5, max: 55, Fig. 1B), corresponding to a median Shannon Index of 1.18 (min: 0.18, max: 2.26, Fig. 1B), reflecting a low richness and evenness within each sample. Unlike this low α-diversity, we observed a high β-diversity, which quantifies the level of dissimilarity between samples, with a median Bray-Curtis dissimilarity index of 0.87 between the subjects (min: 0.02 max: 1.00, Fig. 1C). We also followed the variations occurring overtime during the 2-week period preceding 3GC exposure. The within subjects’ diversity, measured between the samples collected from the same subject at different time, was almost as high as the between subject diversity, with a Bray-Curtis dissimilarity index of 0.75 (min: 0.08, max: 0.99, Fig. 1C; Fig. S2).

10.1128/mbio.02880-22.2 FIG S2 Heatmap of the Bray-Curtis dissimilarity between the 54 fecal samples for ITS1 sequencing data. Each square represents the comparison between two samples. Bray-Curtis dissimilarity values are ranged from 0 to 1, with 0 being the least dissimilar and 1 being the most dissimilar. 1 to 3 samples are analyzed per individual (i) and are grouped together (green boxes). Download FIG S2, TIF file, 1.0 MB. Copyright © 2022 Delavy et al. 2022 Delavy et al. https://creativecommons.org/licenses/by/4.0/ This content is distributed under the terms of the Creative Commons Attribution 4.0 International license.

We quantified the levels of fecal C. albicans in these volunteers by determining the absolute abundance of C. albicans using specific qPCR. We detected C. albicans DNA at least once between −D15 and −D1 in 20/21 subjects (95.2%) before 3GC administration. In total, 42/51 samples analyzed were positive for C. albicans (82.4%). In these samples, C. albicans DNA levels ranged from 2.8 × 10^−4^ to 1.26 ng/g of stool, with a median of 9.4 × 10^−3 ^ng/g of stool (Fig. 1D). In comparison, by using ITS1 sequencing data and culture methods, we could detect C. albicans in only 16/22 (72.7%; Table 1) and in 7/22 (15.8%) subjects, respectively.

### Cefotaxime and ceftriaxone exposure increases the fungal load and disturbs the gut mycobiota composition.

**FIG 2:**
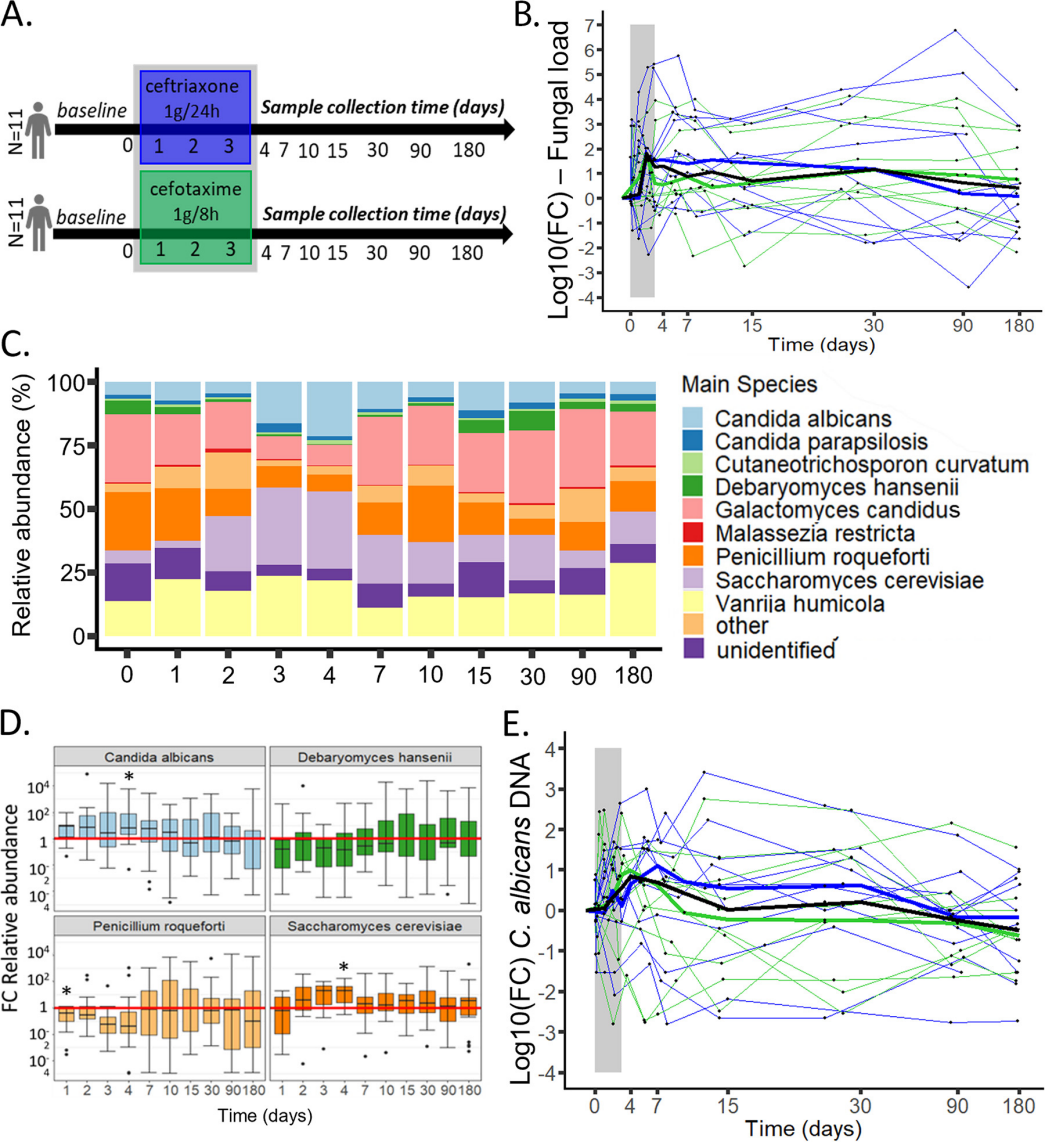
Impact of 3-day cefotaxime and ceftriaxone IV treatment on the gut mycobiota of 22 healthy subjects followed for a 6-month period. (A) Study design. (B) Log_10_ (foldchange [FC]) of the fungal load following ceftriaxone and cefotaxime treatment (gray area). Thin lines represent the subjects, thicker lines represent the medians at each day for each treatment group (blue and green) and for all subjects (black). (C) Main fungal species distribution following ceftriaxone and cefotaxime treatment. (D) Distribution of the relative abundance log_10_ (FC) AUCs for Candida albicans, Debaryomyces hansenii, *Penicillium roqueforti*, and Saccharomyces cerevisiae, highlighting the duration and the amplitude of the perturbations *q value < 0.05, Wilcoxon t-test, false-discovery rate correction. Upper whiskers extend from the hinge to the largest value below 1.5× the interquartile range, and the lower whiskers extend from the hinge to the smallest value above 1.5× the interquartile range. (E) Log_10_ (FC) of C. albicans DNA levels following ceftriaxone and cefotaxime treatment (gray area). Thin lines represent the subjects, thicker lines represent the medians at each day for each treatment group (blue and green) and for all subjects (black).

To measure how much the antibiotic treatment affected the gut mycobiota, we compared its features, including the fungal load, genera and species composition, and C. albicans absolute levels, at baseline (D0) with those during and after antibiotics (Fig. 2A). Data collected at −D1 were used as baseline. If missing, −D7 data were used instead (see Fig. S3).

10.1128/mbio.02880-22.3 FIG S3 Mycobiota characteristics at baseline (D0) for 22 healthy subjects. (A) Violin plot of the fungal load observed among the 22 healthy subjects' samples collected at baseline. (B) Boxplot of the main fungal species the relative abundance observed among the 22 healthy subjects at baseline. (C) Alpha diversity: violin plot of the number of OTUs and of the Shannon index values observed among the 22 healthy subjects’ samples collected at baseline. (D) Violin plot of the C. albicans DNA concentrations observed among the 22 healthy subjects’ samples collected at baseline. For all figures, the upper whiskers extend from the hinge to the largest value below 1.5× the interquartile range, and the lower whiskers extend from the hinge to the smallest value above 1.5× the interquartile range. Download FIG S3, TIF file, 0.9 MB. Copyright © 2022 Delavy et al. 2022 Delavy et al. https://creativecommons.org/licenses/by/4.0/ This content is distributed under the terms of the Creative Commons Attribution 4.0 International license.

We used two metrics to estimate changes during and after antibiotic administration: the areas under the curve (AUCs) of the mycobiota characteristics’ changes from D0, and the changes from D0 of the mycobiota characteristics, for each subject, at different time points between D1 and D180. The first metric allows the aggregation of both the duration of the changes and their amplitude whereas the second allows the detection of more punctual variations.

We observed a general long-term increase of the fungal load in the 22 subjects early after the start of the antibiotic treatment. The fungal load significantly increased immediately after the start of antibiotics, independently of the antibiotic used, with a positive AUCs for all calculated periods between D0 and D2, and D0 and D90 (Wilcoxon test; *P* values of 0.008, 0.017, 0.040, 0.014, 0.009, 0.005, 0.006, and 0.048, respectively) with a maximal 62.3-fold increase at D2 (min: 0.02, max: 1.8 ×10^4^; Wilcoxon test; *P* value of 0.007; Fig. 2B; Fig. S4B; Table S1). No difference was observed between the subjects treated with ceftriaxone and those treated with cefotaxime (Fig. 2B; Table S2).

10.1128/mbio.02880-22.4 FIG S4 Impact of the antibiotic treatment on the gut mycobiota genera composition. (A) Distribution of the relative abundance log_10_ (FC) AUCs for *Candida* sp., *Debaryomyces* spp., *Penicillium* sp., and *Saccharomyces* sp., highlighting the duration and the amplitude of the perturbations. (B) Distribution of the relative abundance foldchanges (FC) *Candida* sp., *Debaryomyces* sp., *Penicillium* sp., and *Saccharomyces* sp., highlighting punctual perturbations. (C) Distribution of the relative abundance log_10_ (FC) AUCs for Candida albicans, Debaryomyces hansenii, *Penicillium roqueforti*, and Saccharomyces cerevisiae, highlighting the duration and the amplitude of the perturbations. For all figures, the upper whiskers extend from the hinge to the largest value below 1.5× the interquartile range, and the lower whiskers extend from the hinge to the smallest value above 1.5× the interquartile range. *q value < 0.05, Wilcoxon t-test, false-discovery rate correction. Download FIG S4, TIF file, 2.0 MB. Copyright © 2022 Delavy et al. 2022 Delavy et al. https://creativecommons.org/licenses/by/4.0/ This content is distributed under the terms of the Creative Commons Attribution 4.0 International license.

10.1128/mbio.02880-22.5 TABLE S1 Weighted areas under the curves (AUC) and changes from baseline of the mycobiota features. Download Table S1, XLSX file, 0.02 MB. Copyright © 2022 Delavy et al. 2022 Delavy et al. https://creativecommons.org/licenses/by/4.0/ This content is distributed under the terms of the Creative Commons Attribution 4.0 International license.

10.1128/mbio.02880-22.6 TABLE S2 Weighted areas under the curves (AUC) and changes from baseline of the mycobiota features according to the treatment group. Download Table S2, XLSX file, 0.02 MB. Copyright © 2022 Delavy et al. 2022 Delavy et al. https://creativecommons.org/licenses/by/4.0/ This content is distributed under the terms of the Creative Commons Attribution 4.0 International license.

At D15, we observed a slight increase of the number of fungal OTUs, compared with D0 (Wilcoxon test; *P* value of 0.030; Table S1) but not of the Shannon Index (Wilcoxon test; *P* value of 0.47), suggesting that the fungal α-diversity is not strongly impacted by the antibiotics. No difference was observed between the subjects treated with ceftriaxone or those treated with cefotaxime, and this for all fungal diversity indices studied (Table S2).

Three genera were significantly impacted by the antibiotics *Debaryomyces* sp., *Penicillium* sp., and *Saccharomyces* sp. (Fig. S4A and B; Table S1). *Debaryomyces* sp. were significantly decreased immediately after the start of the treatment, with negative AUCs between D0 and D3 (Wilcoxon test; *q* value of 0.02) and a maximal but not significant 12.5-fold drop at D3 (min: 0.09, max: 1.7 × 10^5^, Wilcoxon test; *q* value of 0.08). *Penicillium* sp. were also decreased immediately after the start of the treatment, with negative AUCs between D0 and D2 and up to D0 and D7 (Wilcoxon test; *q* values of 0.01, 0.005, 0.003, and 0.0002, respectively) with a maximal 21.4-fold decrease at D4 (min: 0.81, max: 776.2; Wilcoxon test; *q* value of 0.0008). On the contrary, *Saccharomyces* sp. relative abundance was punctually increased at D4 (median: 19.5-fold increase, min: 0.32, max: 169.8), compared with baseline (Wilcoxon test; *q* value of 0.01), before returning to basal levels. No significant difference between the subjects of the two treatment groups was observed at any day, for all genera tested (Table S2).

In addition, at the species level, four taxa were significantly affected by 3GC treatment: S. cerevisiae, *D. hansenii*, *P. roqueforti*, and C. albicans (Fig. 2C; Table S1). *D. hansenii* was decreased for the period D0 to D3, with a corresponding negative AUC (Wilcoxon test; *q* value of 0.047; Fig. S4C) and *P. roqueforti* was punctually reduced after the treatment with a 2.4-fold drop at D1 (min: 0.74, max: 3.3 × 10^3^; Wilcoxon test; *q* value of 0.026; Fig. 2D). In contrast, C. albicans and S. cerevisiae relative abundance displayed a 9.8-fold and 19.5-fold raise at D4, respectively (C. albicans*:* max: 1.1 × 10^5^, min: 0.004; Wilcoxon test; *q* value of 0.04, S. cerevisiae*:* max: 169.8, min: 0.32 Wilcoxon test; *q* value of 0.026; Fig. S4C). As for the genera, no significant difference between the subjects of the two groups was observed for any species (Table S2).

Not only C. albicans relative abundance but also its absolute abundance was punctually increased after antibiotics. Indeed, 3GC administration led to a punctual raise of C. albicans DNA levels on the D0 to D4 period (Wilcoxon test, *q* value of 0.047) with a maximal 2.1-fold increase at D2 (min: 0.03, max: 288.4; Wilcoxon test; *P* value of 0.02; Fig. 2E), when measured by qPCR. However, this increase of C. albicans DNA levels was subject-dependent. For example, subject 1 displayed an impressive increase of C. albicans DNA, with a maximal 2,521.3-fold raise at D15 whereas C. albicans DNA levels were reduced in subject 12 after the treatment. No difference was observed between the two groups of treatment at any days (Fig. 2E; Table S2).

### Change in β-lactamase activity levels as a key parameter for C. albicans overgrowth in the GI tract after third-generation cephalosporin administration.

**FIG 3:**
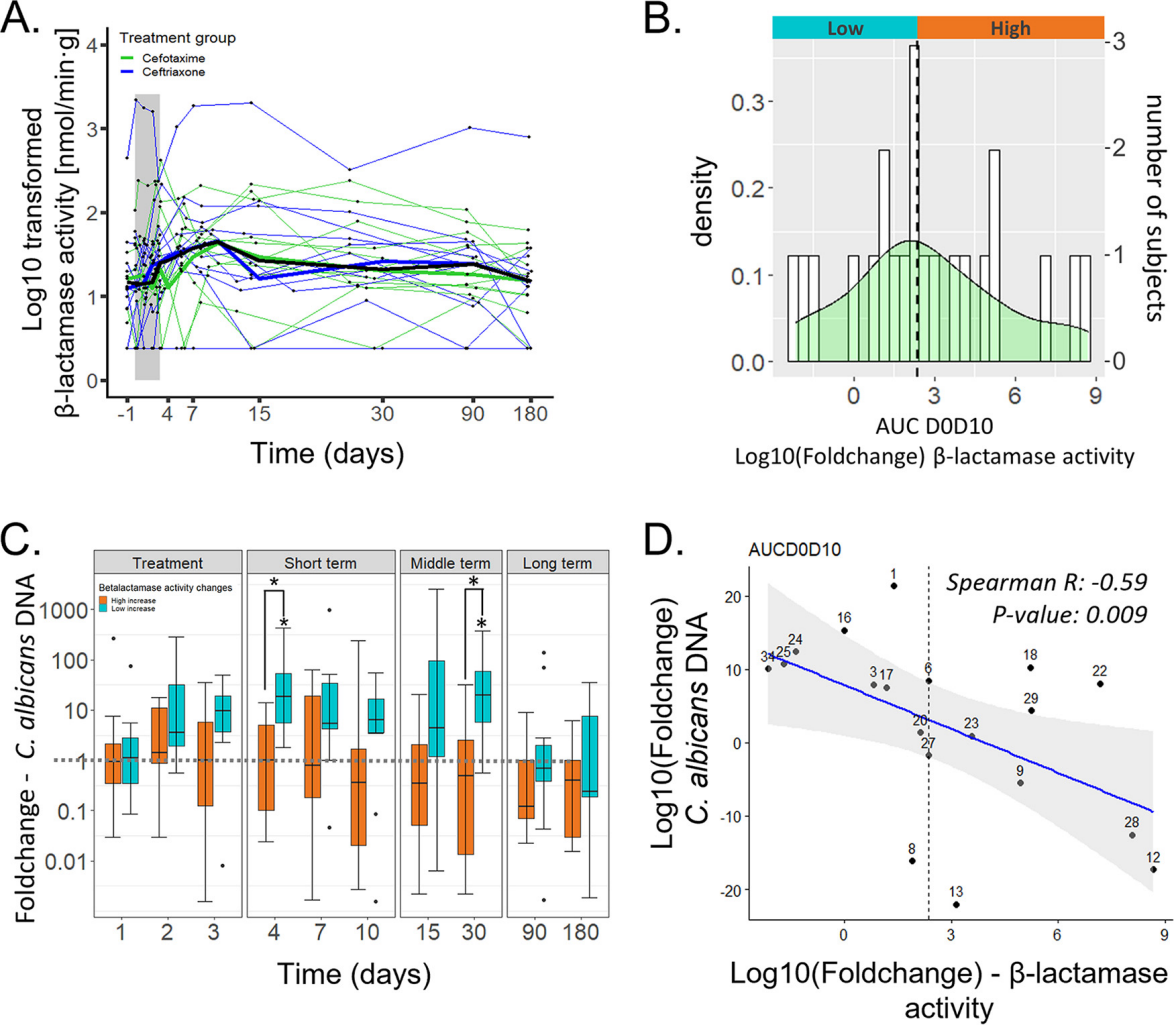
Change in β-lactamases activity levels as a key parameter for C. albicans proliferation in the gut after third-generation cephalosporin administration. (A) β-lactamases activity before, during, and after the antibiotic treatment. Thin lines represent the subjects; thicker lines represent the medians at each day for each treatment group (ceftriaxone, blue; cefotaxime, green) and for all subjects (black). (B) Distribution of the D0 to D10 AUCs of the change of β-lactamases activity in 22 healthy subjects. The density distribution is represented by the green curve and the number of subjects for each range of AUC values are represented by the white histogram. The group “high” (orange) regroups the subjects with a D0 to D10 AUC above the median (black dashed line) and the group “low” (blue) regroups the subjects with a D0 to D10 AUC below the median. (C) Boxplots of C. albicans DNA levels log_10_ (FC) following ceftriaxone and cefotaxime treatment. Orange boxplots indicates values for the subjects from the group “high” of and blue boxplots represents the values for the subjects from the group “low.” Upper whiskers extend from the hinge to the largest value below 1.5× the interquartile range, and the lower whiskers extend from the hinge to the smallest value above 1.5× the interquartile range. (D) Correlation plot of the log_10_ (FC) AUCs of C. albicans DNA levels and β-lactamases activity for the D0 to D10 period. Regression is represented by a blue line and the confidence interval by the gray area. Subjects are designated by their ID number.

β-lactamase activity was measured in each fecal sample by dosing the NFC-hydrolyzing activity. This activity was heterogenous between subjects before antibiotics, ranging from 2.40 to 1,240 nmol/min·g of stool, with no difference between the two groups that received either ceftriaxone or cefotaxime (Wilcoxon test, *P* value: 0.78). Globally, β-lactamase activity was significantly increased after 3GC administration for all the periods calculated between D0 to D3 and D0 to D180 (Wilcoxon test; *P* values of 0.040, 0.006, 0.002, 0.0008, 0.0003, 0.0006, 0.0007, and 0.03, respectively), with a maximal 2.25-fold increase at D7 (Fig. 3A). However, we observed two types of behavior in the D0 to D10 AUC of the change in β-lactamase activity, with some subjects displaying a high increase of the β-lactamase activity after antibiotic treatment (up to a 28-fold rise) whereas others showed no change or even a decrease of this activity (up to a 7-fold decrease). Therefore, we split the 22 subjects in two groups; the “high” group was characterized by a strong increase of the fecal β-lactamase activity (AUC D0 to D10 ≥ 2.36), whereas the “low” group had a lower increase or even a decrease of this activity (AUC D0 to D10 < 2.36; Fig. 3B). Changes of C. albicans DNA levels were significantly different between subjects of the “low” and “high” groups, both for the D0 to D10 period and at D4 and D30 (Wilcoxon test; *P* value of 0.02, 0.02, and 0.007, respectively). At D4 and D30, C. albicans DNA levels were significantly increased in the group “low,” whereas no change was detected in the group “high” (D4: Wilcoxon test; *P* value of 0.008 and 0.84, respectively; D30: Wilcoxon test; *P* value of 0.023 and 0.26, respectively; Fig. 3C).

Finally, we showed a highly significant negative interaction between the D0 to D10 AUC of the change in β-lactamase activity and the D0 to D10 AUC of the change in C. albicans DNA levels (Spearman correlation; R: −0.59, *P* value: 0.009; Fig. 3D). No such correlation could be found between the D0 to D10 AUC of the fungal load and the D0 to D10 AUC of the change in β-lactamase activity (Spearman correlation; R: −0.25, *P* value: 0.3).

## DISCUSSION

In this study, we explored the impact of β-lactam antibiotics on the human gut mycobiota by performing a targeted metagenomic analysis of the mycobiota of healthy subjects before, during, and after 3GC exposure. 3GC strongly affected the mycobiota, especially C. albicans carriage, with wide intersubject variations that were not related to the type of 3CG they received. We identified the changes of fecal β-lactamase activity after treatment as a potential key factor regulating C. albicans overgrowth, with subjects characterized by a low increase of β-lactamase activity displaying a stronger increase of C. albicans levels following antibiotics. This regulation is likely mediated by a differential impact of antibiotics on the endogenous gut bacteria, according to differences in the occurrence of β-lactamase-producing bacteria in the microbiota. Briefly, a microbiota rich in β-lactamase-producing bacteria would favor 3GC hydrolysis, reduced antibiotic-induced microbiota dysbiosis, and reduced C. albicans overgrowth. In contrast, a microbiota poor in β-lactamase-producing bacteria would allow 3GC maintenance, high antibiotic-induced microbiota dysbiosis, and high C. albicans overgrowth. This phenomenon may explain the so-called C. albicans colonization resistance experienced by some individuals. Such colonization resistance has been the subject of an old and preliminary report (11) but has not been further explored until this present study. Overall, these results are coherent with the hypothesis stating that specific intestinal bacteria or their metabolites regulate C. albicans overgrowth (6, 14, 15, 18, 19). Our results attest that the same antibiotic regimen may affect differentially the microbiota and consequently lead to different risks of C. albicans overgrowth depending on the subject that receives it. The current paradigm stating that antibiotics are systematically a risk factor for C. albicans overgrowth should thus be adjusted for treatments based on β-lactams antibiotics. Monitoring fecal β-lactamase activity during and after a β-lactams antibiotic treatment could be an accurate predictor of the actual risk of a later increase of C. albicans burden.

As importantly, we found that C. albicans was present, in varying quantity, in the gut of almost every healthy subject. This study is the first to use a qPCR method to quantify and follow C. albicans carriage, allowing an increasingly specific detection. Using more traditional assays, the prevalence of C. albicans in these subjects was much lower and close to what has been previously reported (20–22). This suggests that our results reflect the reality of what is the true presence of C. albicans in the gut of healthy humans. If confirmed in a larger study, this might indicate that C. albicans is not a facultative commensal as previously thought, but that it is able to maintain itself in the gut of most individuals, even at very low concentration.

That almost all subjects in this study were colonized by C. albicans renders our cohort particularly adapted to follow the effects of antibiotic treatment on C. albicans carriage. Moreover, contrary to what has been done in other studies (6), our focus on a single antibiotic family allows a precise understanding on how 3GC, a largely used antibiotics family, acts on the human gut mycobiota. This allowed us to show that 3GC strongly affect the gut mycobiota, with a global increase of the fungal load, as well as a punctual perturbation of several fungal species and genera, including C. albicans. Indeed, both C. albicans relative and absolute abundances were increased after the start of antibiotics. This is particularly concerning since a recent report showed that the administration of β-lactam antibiotics leads to increased virulence of C. albicans (23). By killing Gram-negative commensal bacteria, β-lactams cause the release of a large amount of peptidoglycans, which can then induce C. albicans hyphal growth, an essential virulence factor of this species (23). Moreover, a recent study showed that exposure to non-3GC broad-spectrum antibiotics not only promotes susceptibility to C. albicans systemic infection in mice, but also increases the mortality, through an impairment of the lymphocyte-dependent IL-17A- and GM-CSF-mediated response (24). Therefore, C. albicans cannot only growth in patients treated with 3GCs, but its disruptive abilities might also be increased. This can be particularly problematic, not only for immunosuppressed patients, but also for those with inflammatory bowel disease that are already carrying higher levels of C. albicans (25, 26) in their gut.

Overall, most of the mycobiota perturbations following 3GC treatment were subject-dependent, with some subjects more impacted than others. This is not particularly surprising considering the within- and between-subjects Bray-Curtis dissimilarity observed pretreatment. This has already been reported (21) and suggests that the largest part of the fecal mycobiota is made of transient species brought by the diet, such as *D. hansenii*, which is commonly found in cheese (27), or potentially by the respiration of spores of filamentous fungi, which can then be swallowed, such as *Penicillium* sp. This hypothesis is supported by a recent study showing that diet-associated fungi are recovered with low relative abundances in mucosal surgical-recovered samples, highlighting the differences observed in the gut mycobiota depending on its spatial organization across the GI tract (28). Moreover, associated fungi strongly contribute to the fungal biomass of the fecal microbiota (29). Our results would also indicate that contrary to *Penicillium* sp. or *D. hansenii*, C. albicans main reservoir is indeed humans, which would explain why an environmental reservoir for this species has yet to be found (18, 30, 31).

Finally, fungi represented only a small fraction of the total microbiota based on total and fungal DNA quantification. This underrepresentation of the fungal community in the human gut has already been reported (32), but the authors did not quantify the exact proportion of the mycobiota. More recently, the fungal load of 24 healthy subjects was estimated, with results very similar to ours (33). Finally, Doron et al. confirmed that the fungal biomass was low within the gut microbiota, representing only 1% to 2% of the microbial biomass of the gut (29). However, to our knowledge, this present study is the first to assess the day-to-day variation of the fungal load in healthy individuals.

Taken together, this study offers a better understanding of the factors behind C. albicans overgrowth after antibiotics. We showed that a same antibiotic treatment may disturb differentially the gut microbiota, depending on the subject that receives it. This highlights the importance of a more personalized use of antifungal prophylaxis, and helps limiting the selection of fungi resistant to antifungal drugs in patients at high risk of invasive candidiasis, such as intensive care unit or haemato-oncology patients.

## MATERIALS AND METHODS

### CEREMI cohort.

In this study, we used fecal samples from the CEREMI study, a prospective, open-label, and randomized clinical trial conducted from March 2016 to August 2017 in healthy adult subjects at the Clinical Investigation Center at Bichat-Claude Bernard Hospital (Paris, France) (8). Participants were given oral and written information and had to return signed consent before inclusion in the trial. For more information about the clinical trial, see Burdet et al. (8).

The 22 included subjects were randomized in a 1:1 ratio and were treated for 3 days with either ceftriaxone (1 g/24 h) or cefotaxime (1 g/8 h). 3GC were administered as a 30-min intravenous infusion using an automatic high-precision infusion pump.

Fecal samples were collected before treatment at −D15, −D7, and −D1; during treatment at D1, D2, and D3; and after treatment at D4, D7, D10, D15, D30, D90, and D180. Fecal samples were stored at −80°C.

### Fungal DNA extraction from fecal samples.

For each sample, 250 mg of stool was processed following the repeated bead beating plus column method described by Yu and Morrison (34), except than a FastPrep-24 device (MP Biomedicals, Belgium) was used instead of a Mini-Beadbeater.

Total fecal DNA levels were measured by Qubit (Invitrogen, USA) using the dsDNA Broad Range Kit (Invitrogen, USA). Samples for which this concentration was below 50 ng/μL were excluded from the analysis.

### ITS1 sequencing.

We prepared amplicon libraries, targeting the ITS1 region, using ITS1F and ITS2 primers (35, 36). Amplicon were generated by PCR using a 96-well thermal cycler in the following conditions: 95°C for 3 min, 25 cycles of 95°C for 30 s, 55°C for 30 s, 72°C for 30 s, and 72°C for 5 min, and cooling at 4°C. Amplicons were purified with AMPure XP (Beckman Coulter, USA) as described in the 16S Metagenomic Sequencing Library Preparation guide (37). Adapter were attached using Nextera XT Index Kit (Illumina, France) and the index PCRs were performed in the following conditions: 95°C for 3 min, eight cycles of 95°C for 30 s, 55°C for 30 s, 72°C for 30 s, and 72°C for 5 min, and cooling at 4°C. Barcoded PCR products were purified with AMPure XP (Beckman Coulter, USA) and verified and quantified on a Bioanalyzer DNA 1000 chip (Agilent, USA). Samples were normalized at 4 nM and pooled into a library, using 5 μL of each diluted sample. A PhiX sequencing control was prepared following the manufacturer’s instructions. The libraries were sequenced in 300-bp paired-end using the MiSeq reagent kit V3 on Illumina MiSeq platform (Illumina, Evry, France).

### OTU building process and taxonomic assignment.

We recovered 8,819,635 amplicons from ITS1 region. After removal of singletons and chimera amplicons using the SHAMAN pipeline (38), we clustered the 56,634 remaining amplicons in OTUs using a cut-off value of 97% similarity. Thus, 4,648 OTUs were obtained and 551 of them could be aligned against fungal sequences of the UNITE database. On these 551 fungal OTUs, 340 were present in at least two fecal samples and were conserved for the downstream analyses. We performed a first round of annotation on SHAMAN against the UNITE database (rev. 8.0) and then a second round against a more recent release of UNITE (rev. 8.2). The OTUs we could not annotate were submitted to a classic BLASTN. Only hits matched with a similarity above 97% to reference genomes were conserved. The abundances and weighted non-null normalized counts tables were generated with SHAMAN (38).

### Quantitative PCR for detection of total fungal load in human DNA samples.

Fungal DNA was quantified by TaqMan qPCR as described by Liu et al. (39) using a double dye MGB 5′ 6-FAM-labeled probe (Eurogentec, Belgium). All reactions were performed on a CFX96 real-time PCR system (Bio-Rad, USA) with the following conditions: 2 min at 50°C, 10 min at 95°C, 15 s at 95°C, and 1 min at 65°C, the last two steps repeated for 45 cycles. All samples were tested in two independent rounds, each time in duplicates.

The fungal load was estimated by dividing the fungal DNA concentration by the total DNA concentration of the sample (33), obtained by Qubit Broad Range protocol.

### Quantitative PCR for detection of C. albicans DNA in human DNA samples.

At 1:10 dilution, 7.5 μL of the extracted total fecal DNA were used as a template for TaqMan qPCR analysis, using probe and primers described by Guiver et al. (40), at 0.1 μM and 0.2 μM, respectively. All reactions were performed on a CFX96 real-time PCR system (Bio-Rad, USA) with the following conditions: 2 min at 50°C, 10 min at 95°C, 15 s at 95°C, and 1 min at 62°C; the last two steps repeated for 45 cycles. All samples were tested in two independent rounds, each time in duplicates.

### Quantitative PCR amplification control.

To exclude the presence of qPCR inhibitors, samples diluted at 1:10, were submitted to the Universal Exogenous qPCR Positive Control for TaqMan Assay (Eurogentec, Belgium), using a Cy5-QXL 670 Probe system (Eurogentec, Belgium). Manufacturer’s recommendations were followed.

### Culture of the fecal samples.

A 10-μL loop of fecal samples was mixed with 100 μL H_2_O and plated on a *Candida* CHROMAGAR medium plate (bioMérieux, France). Potential C. albicans colonies were further tested by MALDI-TOF MS (Brucker, USA) to confirm the identification.

### Measure of the β-lactamase activity.

Fecal β-lactamase activity was quantified by measuring the hydrolysis of nitrocephin, a chromogenic cephalosporin. Activity was measured at least in duplicate.

Fecal samples (stored at 65°C) were thawed 30 min on ice. Then, 140 mg to 380 mg of each fecal sample was mixed with 5 mL/g of stool HZn buffer (50 mM (2-hydroxyethyl)-1-piperazineethanesulfonic acid (HEPES) buffer, pH 7.5, supplemented with 50 μM ZnSO4) and agitated for 1 h. Samples were centrifuged twice at 4°C (15 min and 30 min). Then, 3 to 20μL of the obtained supernatant were mixed with 100 μM nitrocefin (Cayman Chemical, USA) and HZn buffer to reach a final volume of 200 μL. Samples were incubated 20 min at room temperature in a 1:1 ratio with HZn buffer. Nitrocefin hydrolysis was monitored in EnVision microplate reader (Perkin Elmer, USA) at a 482-nm wavelength. SpectraPlate-96 (Perkin-Elmer, USA) using an automated liquid handling Janus Integrator system (Perkin Elmer, USA) was used to conduct the assays.

β-lactamase activity was normalized to one gram of fecal sample and to 1-cm pathlength. Detection threshold was set at a cut-off value of 4.8 nmol/min·g of fecal sample.

### Biostatistical analyses.

All analyses were performed on R (version 4.0.2 [41]). We used the vegan package (v.2.5-6 [42]) to compute diversity indexes and ggplot2 package to generate the figures (v. 3.3.5 [43]).

We used samples collected at −D1 before treatment as baseline, called D0. If this sample was missing for a subject, sample collected at −D7 before treatment was used instead. If both samples were missing, sample collected at −D15 before treatment was used as D0 sample.

We calculated the change from baseline of the fungal load, C. albicans DNA absolute abundance, the relative abundance of the fungal genera, and species and β-lactamase activity. Null values were replaced by the minimal non-null value of the given variables divided by two, to allow a log_10_ transformation. Only the fungal genera and species reaching a maximal relative abundance superior to 1% for at least half of the subjects were analyzed. We calculated the AUCs using the R package MESS (v. 0.5.7, [44]) for each period from D0 to D2 up to D0 to D180 based on the normalized changes from baseline and the actual time and date of stool collection.

For all analyses, we used bilateral nonparametric Wilcoxon exact tests. We used a type I error of 0.05 and corrected the *P* values for multitesting using false discovery rate correction.

### Data availability.

The data sets generated during and/or analyzed during the current study are available from the corresponding author on reasonable request.
