# Supplementary figures and images for: A Clinical Study Provides the First Direct Evidence That Interindividual Variations in Fecal β-Lactamase Activity Affect the Gut Mycobiota Dynamics in Response to β-Lactam Antibiotics
Source: mBio. 2022 Nov 30;13(6):e02880-22. doi: 10.1128/mbio.02880-22 (PMC9765473; doi:10.1128/mbio.02880-22)

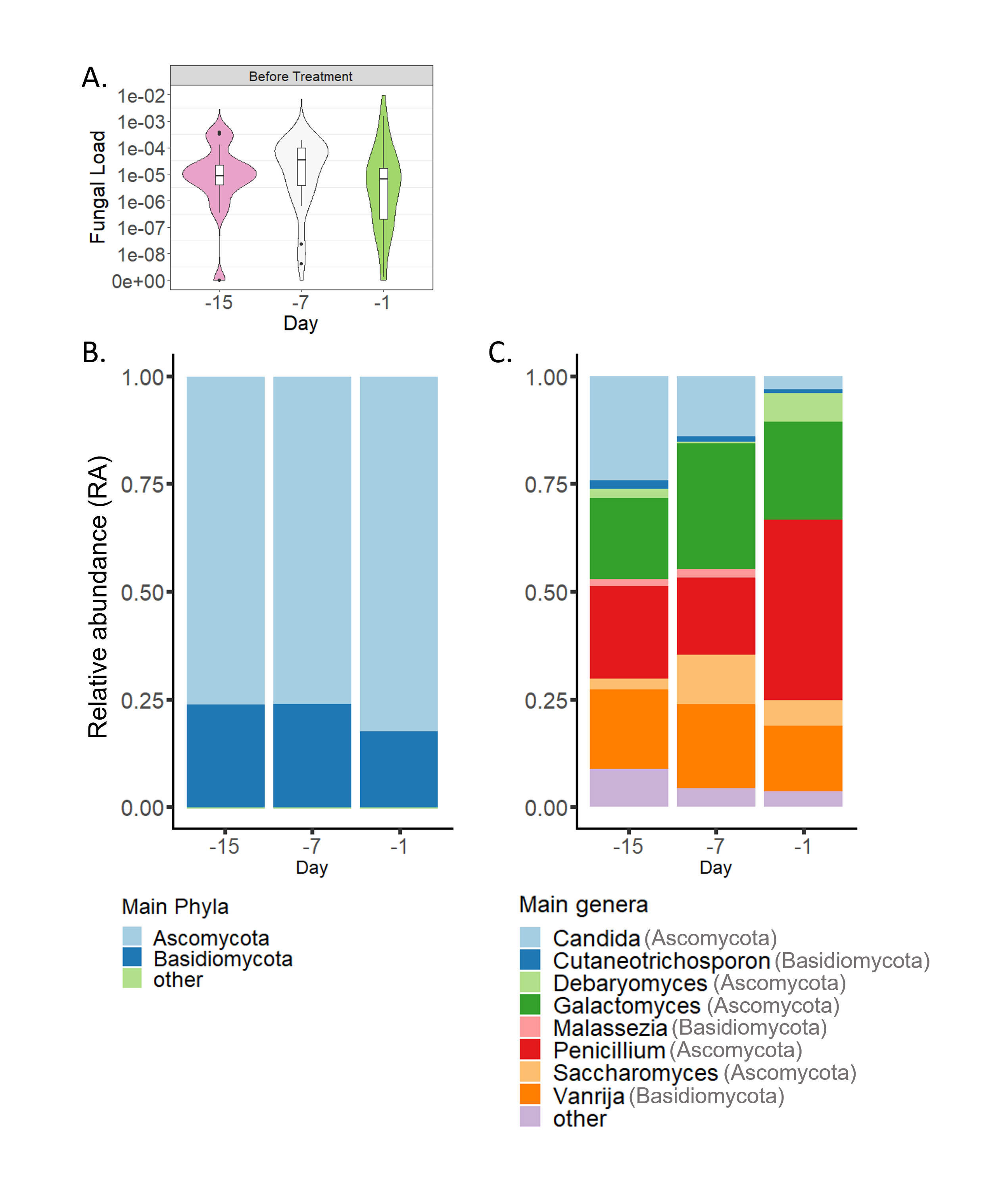

Supplement: FIG S1 [file mbio.02880-22-s0001.tif]

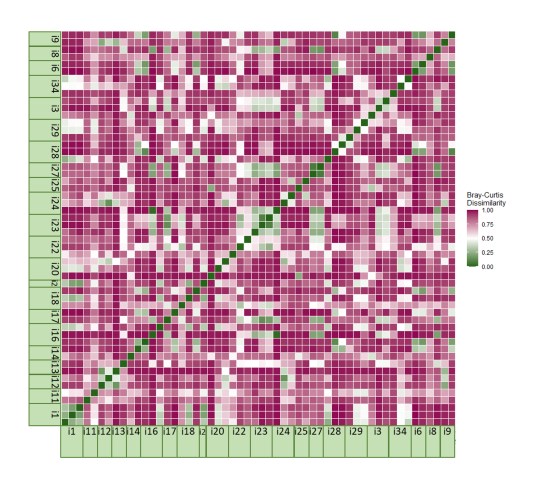

Supplement: FIG S2 [file mbio.02880-22-s0002.tif]

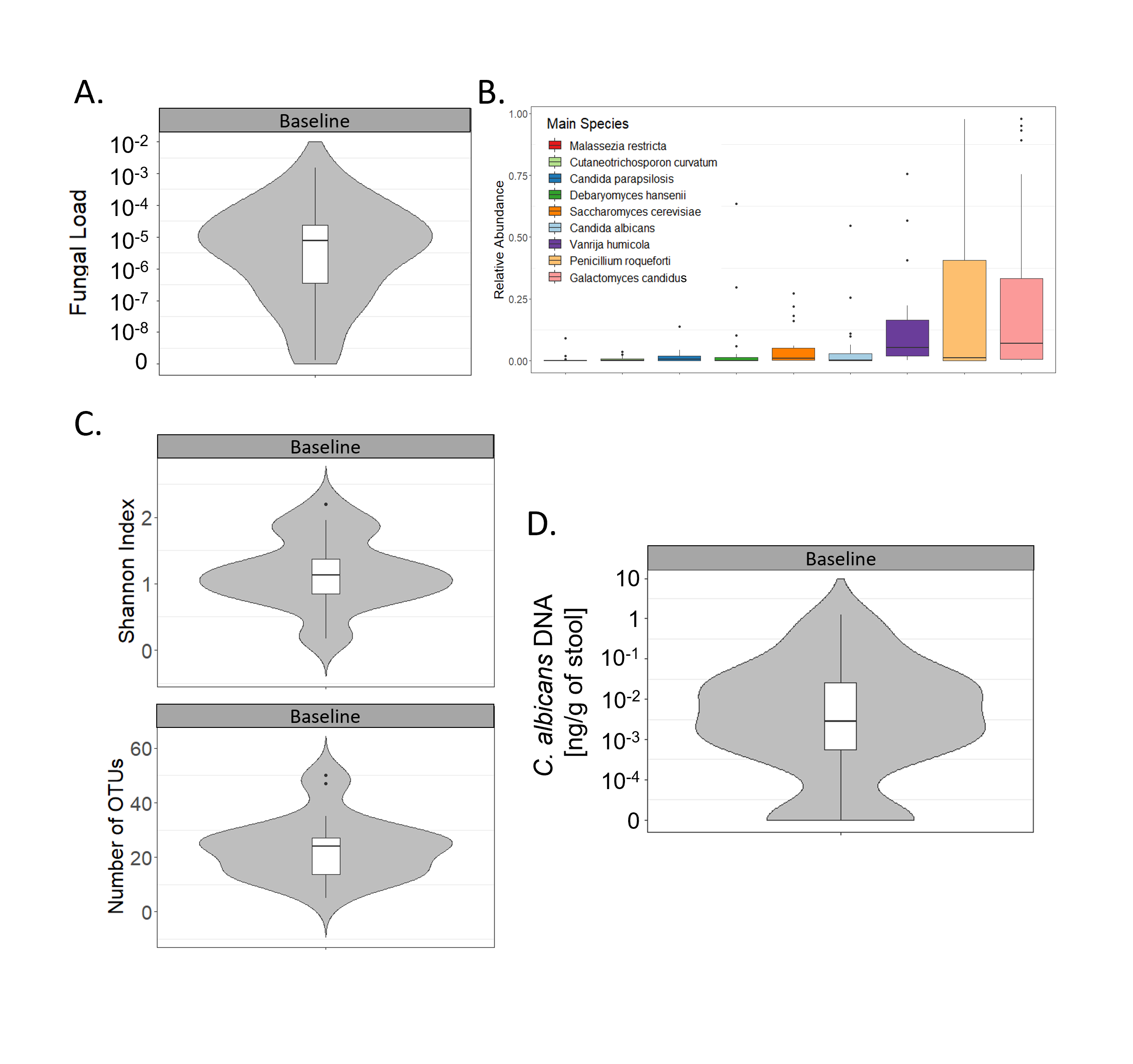

Supplement: FIG S3 [file mbio.02880-22-s0003.tif]

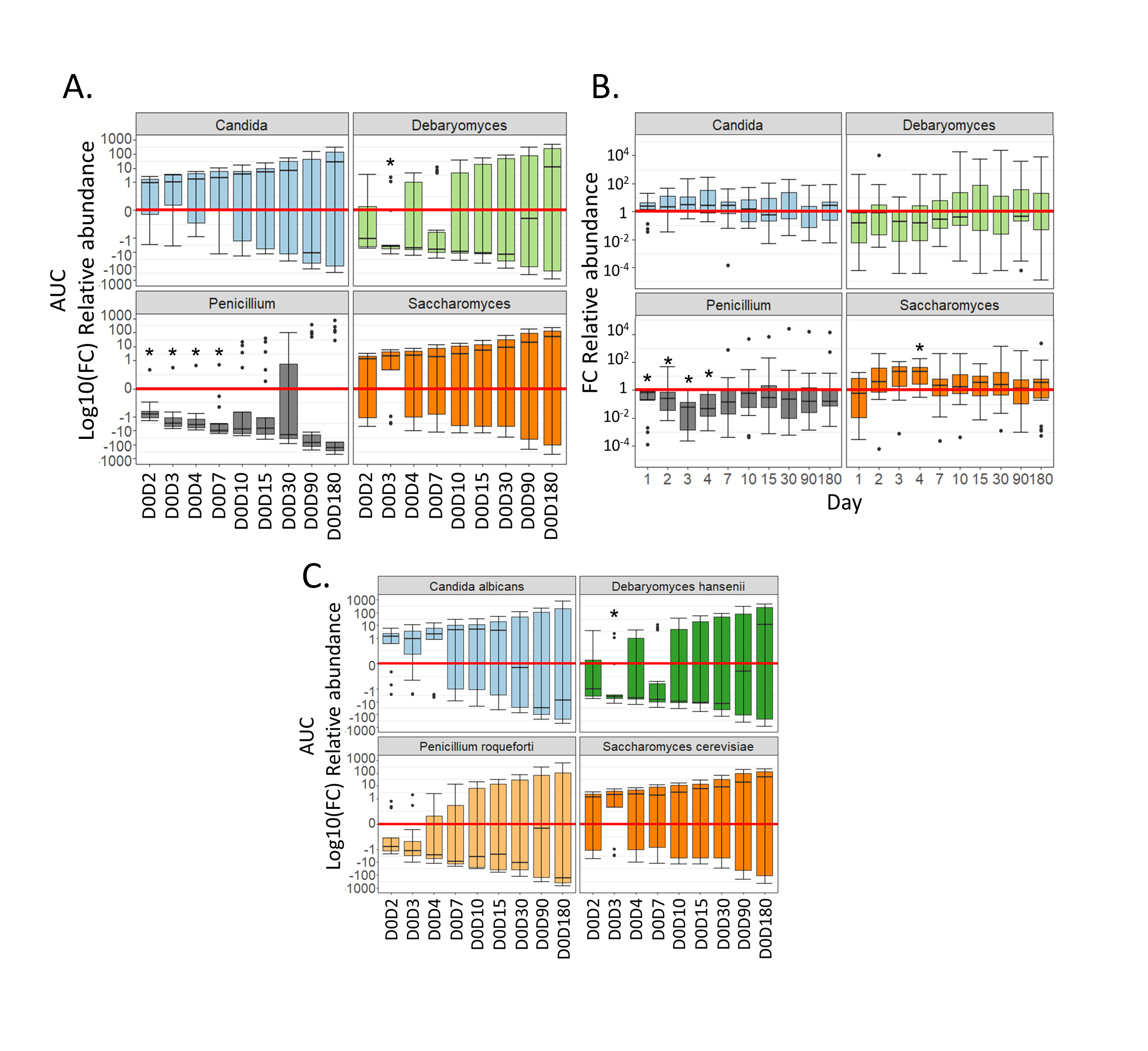

Supplement: FIG S4 [file mbio.02880-22-s0004.tif]
